# Required distal mesorectal resection margin in partial mesorectal excision: a systematic review on distal mesorectal spread

**DOI:** 10.1007/s10151-022-02690-1

**Published:** 2022-08-29

**Authors:** A. A. J. Grüter, A. S. van Lieshout, S. E. van Oostendorp, J. C. F. Ket, M. Tenhagen, F. C. den Boer, R. Hompes, P. J. Tanis, J. B. Tuynman

**Affiliations:** 1 Amsterdam UMC, Vrije Universiteit Amsterdam, Department of Surgery, Cancer Center Amsterdam, Amsterdam, The Netherlands; 2 Rode Kruis Ziekenhuis, Department of Surgery, Beverwijk, The Netherlands; 3 Medical Library, Vrije Universiteit, Amsterdam, The Netherlands; 4 Zaans Medical Centre, Department of Surgery, Zaandam, The Netherlands; 5 Amsterdam UMC, University of Amsterdam, Department of Surgery, Cancer Center Amsterdam, Amsterdam, The Netherlands; 6 Department of Surgical Oncology and Gastrointestinal Surgery, Doctor, Erasmus MC, Rotterdam, The Netherlands

**Keywords:** Distal mesorectal spread, Mesorectal cancer spread, Distal mesorectal resection margin, Partial mesorectal excision, PME

## Abstract

**Background:**

The required distal margin in partial mesorectal excision (PME) is controversial. The aim of this systematic review was to determine incidence and distance of distal mesorectal spread (DMS).

**Methods:**

A systematic search was performed using PubMed, Embase and Google Scholar databases. Articles eligible for inclusion were studies reporting on the presence of distal mesorectal spread in patients with rectal cancer who underwent radical resection.

**Results:**

Out of 2493 articles, 22 studies with a total of 1921 patients were included, of whom 340 underwent long-course neoadjuvant chemoradiotherapy (CRT). DMS was reported in 207 of 1921 (10.8%) specimens (1.2% in CRT group and 12.8% in non-CRT group), with specified distance of DMS relative to the tumor in 84 (40.6%) of the cases. Mean and median DMS were 20.2 and 20.0 mm, respectively. Distal margins of 40 mm and 30 mm would result in 10% and 32% residual tumor, respectively, which translates into 1% and 4% overall residual cancer risk given 11% incidence of DMS. The maximum reported DMS was 50 mm in 1 of 84 cases. In subgroup analysis, for T3, the mean DMS was 18.8 mm (range 8–40 mm) and 27.2 mm (range 10–40 mm) for T4 rectal cancer.

**Conclusions:**

DMS occurred in 11% of cases, with a maximum of 50 mm in less than 1% of the DMS cases. For PME, substantial overtreatment is present if a distal margin of 5 cm is routinely utilized. Prospective studies evaluating more limited margins based on high-quality preoperative magnetic resonance imaging and pathological assessment are required.

**Supplementary Information:**

The online version contains supplementary material available at 10.1007/s10151-022-02690-1.

## Introduction

Heald et al. popularized the concept of total mesorectal excision (TME) as curative surgical treatment for rectal cancer [1]. In both open and laparoscopic TME surgery, the 3-year local recurrence rate (LR) is approximately 5% as reported in large randomized trials [2–6]. Although the laparoscopic approach has shown decreased short-term morbidity compared to the open approach, minimally invasive TME is still associated with high rates of permanent colostomy, postoperative morbidity such as anastomotic leakage and long-term bowel, urinary and sexual dysfunction [7, 8].

Partial mesorectal excision (PME), compared to TME, is a less-extensive surgical procedure associated with less morbidity and better long-term functional outcomes. It has been shown to be associated with significantly lower anastomotic leak rates, increased restorative procedure rate, shorter median hospital stay, less long-term bowel dysfunction and better urinary and sexual function [7–9]. Currently, the indication of necessity for PME or formal TME for patients with proximal rectal cancer remains unclear. The recent consensus on magnetic resonance imaging (MRI) definition of the proximal border of the rectum has improved decision-making on the use of neoadjuvant therapy, but still no consensus exist when to perform a formal TME up to the puborectalis muscle or a PME leaving distal mesorectum with more length of the remaining rectal stump in place and still an adequate distal margin. Only a minority of guidelines consider that PME can be performed and the distal margin of the mesorectum proposed is usually 5 cm [10, 11]. There is a need for consensus on whether a PME or TME should be performed avoiding unnecessary functional impairment while maintaining enough distal margin to incorporate all cancer cells in the specimen.

It is important to have a distal mesorectal margin because of the potential presence of distal mesorectal spread (DMS) of the primary rectal cancer, presenting as mesorectal lymph-node metastases, as vascular or perineural invasion or as tumor deposits. The prevalence and extent of DMS are largely unknown, but these data are prerequisites to define a safe distal resection margin. If oncologically safe, preserving as much as (meso)rectum as possible is preferred for better short-term and long-term functional outcomes [7–9, 12].

The aim of this study was to review the reported pattern (presence and distal spread) of tumor cells in the mesorectum as a step toward reaching consensus about the distal resection margin for patients with rectal cancer undergoing PME.

## Materials and methods

This systematic review was reported in accordance with the guidance of the Preferred Reporting Items for Systematic Reviews and Meta-Analyses checklist (PRISMA) and A MeaSurement Tool to Assess systematic Reviews (AMSTAR) 2 was used as a critical appraisal tool [13, 14]. This study is registered in PROSPERO (ID: CRD42020153098) and the review protocol can be accessed.

## Search strategy

Systematic searches were performed from inception in PubMed (up to October 7th 2019) and Embase.com (up to October 16th 2019) with the assistance of a medical information specialist. The full search strategies for both databases are provided in Table 1 and Table 2. The search query included index terms and free-text words for ‘rectal cancer’, ‘mesorectum’ and ‘metastasis’ or ‘seeding’. Conference abstracts from Embase.com were excluded. No limits on publication date were used. Google Scholar (on November 1^st^ 2019) was also used to look for additional references, using the anonymous mode. References of included studies were checked for other eligible studies.

**Table 1:** Search strategy for PubMed (7 October 2019)

Search	Query	Items found
#1	"Rectal Neoplasms" [Mesh:NoExp] OR (cancer[sb] AND ("Rectum"[Mesh] OR rectum[tiab] OR rectal*[tiab]))	85,150
#2	mesorect*[tiab]	3853
#3	(("Neoplasm Metastasis"[Mesh] OR "Neoplasm Invasiveness"[Mesh] OR metast*[tiab] OR micrometast*[tiab] OR seeding*[tiab] OR circulat*[tiab] OR spread*[tiab])	1,168,095
#4	#1 AND #2 AND #3	1107

**Table 2:** Search strategy for Embase.com (16 October 2019)

Search	Query	Items found
#1	‘rectum tumor’/de OR ‘rectum cancer’/de OR ‘rectum carcinoma’/exp OR ((‘neoplasm’/exp OR carcinoma*:ti,ab,kw OR neoplas*:ti,ab,kw OR tumour*:ti,ab,kw OR sarcoma*:ti,ab,kw OR adenocarcin*:ti,ab,kw OR tumor*:ti,ab,kw OR cancer*:ti,ab,kw OR oncolog*:ti,ab,kw OR malignan*:ti,ab,kw OR metasta*:ti,ab,kw OR carcinogen*:ti,ab,kw OR oncogene*:ti,ab,kw OR paraneoplastic:ti,ab,kw OR plasmacytoma*:ti,ab,kw OR carcinosarcoma*:ti,ab,kw) AND (‘rectum’/exp OR rectal*:ti,ab,kw OR rectum:ti,ab,kw))	114,481
#2	‘mesorectum’/exp OR ‘mesorectal fascia’/exp OR mesorect*:ti,ab,kw	6599
#3	‘metastasis’/exp OR ‘tumor invasion’/de OR ‘tumor seeding’/exp OR metast*:ti,ab,kw OR micrometast*:ti,ab,kw OR seeding*:ti,ab,kw OR circulat*:ti,ab,kw OR spread*:ti,ab,kw	1,651,540
#4	#1 AND #2 AND #3	2038
#5	#4 AND (‘conference abstract’/it OR ‘conference paper’/it OR ‘conference review’/it)	667
#6	#4 NOT #5	1371

## Inclusion and exclusion criteria

Studies eligible for inclusion were studied reporting on the presence of malignant cells in the mesorectum in any form in patients with rectal cancer who underwent radical resection. Studies were excluded if they reported solely the intramural spread of rectal cancer, if they on lateral lymph-node metastases instead of mesorectal spread and if they did not describe the localisation of the mesorectal spread in relation to the primary tumor. Reviews or narrative studies, comment letters or non-human studies were also excluded. In case of studies with suspected overlap of patients, the most recent study was included.

## Selection process

After removal of duplicates, two reviewers (AG and SvO) independently selected the articles by screening on title and abstract using Rayyan QCRI (www.rayyan.ai) [15]. Discrepancies between the two reviewers were resolved by discussion and mutual agreement. If necessary, a third author was consulted in case of disagreement. Thereafter, the same two reviewers independently assessed the resulting articles in full text. References of the included studies were checked to identify further relevant studies.

## Quality assessment and scoring

The Agency for Healthcare Research and Quality (AHRQ) recommends 11 items to assess methodological quality of cross-sectional studies [16, 17]. That checklist was used for the included studies. Every item was judged with the use of “Yes”, “No”, “Unclear” or “Not applicable”. If there was any discrepancy between the two reviewers, it was resolved by discussion and two-way agreement. If required, a third author was consulted to reach an agreement. There were no consequences for low-quality studies, because there are no abundance of data.

## Outcomes of interest

Primary outcome was the distance of tumor cells in the mesorectum distal from the rectal tumor. All kinds of malignant spread in the mesorectum, in form of lymph nodes, tumor deposits, direct invasion or lymphatic permeation and extramural vascular invasion (EMVI), were extracted. The participants with DMS were classified in different subgroups depending on characteristics of the primary rectal cancer and whether or not long-course neoadjuvant chemoradiotherapy (CRT) was given. We intended to provide the DMS for every clinical distinct subgroup of rectal cancer based on, for example, TNM stage and tumor height. Low rectal cancer was defined as the lower edge of the tumor < 7 cm from the anal verge and high rectal cancer as a distance of ≥ 7 cm from the anal verge.

## Quantitative analysis

Data analysis was performed using IBM SPSS Statistics 26. The mean and maximum DMS were calculated and displayed in tables and figures. The mean DMS was calculated only from patients where the distance of the DMS relative to the tumor was reported, so the primary data of the articles were used to calculate the means. In addition, two scatter plots were constructed to illustrate the distribution of DMS per subgroup of rectal cancer.

## Results

### Literature search

**Fig. 1:**
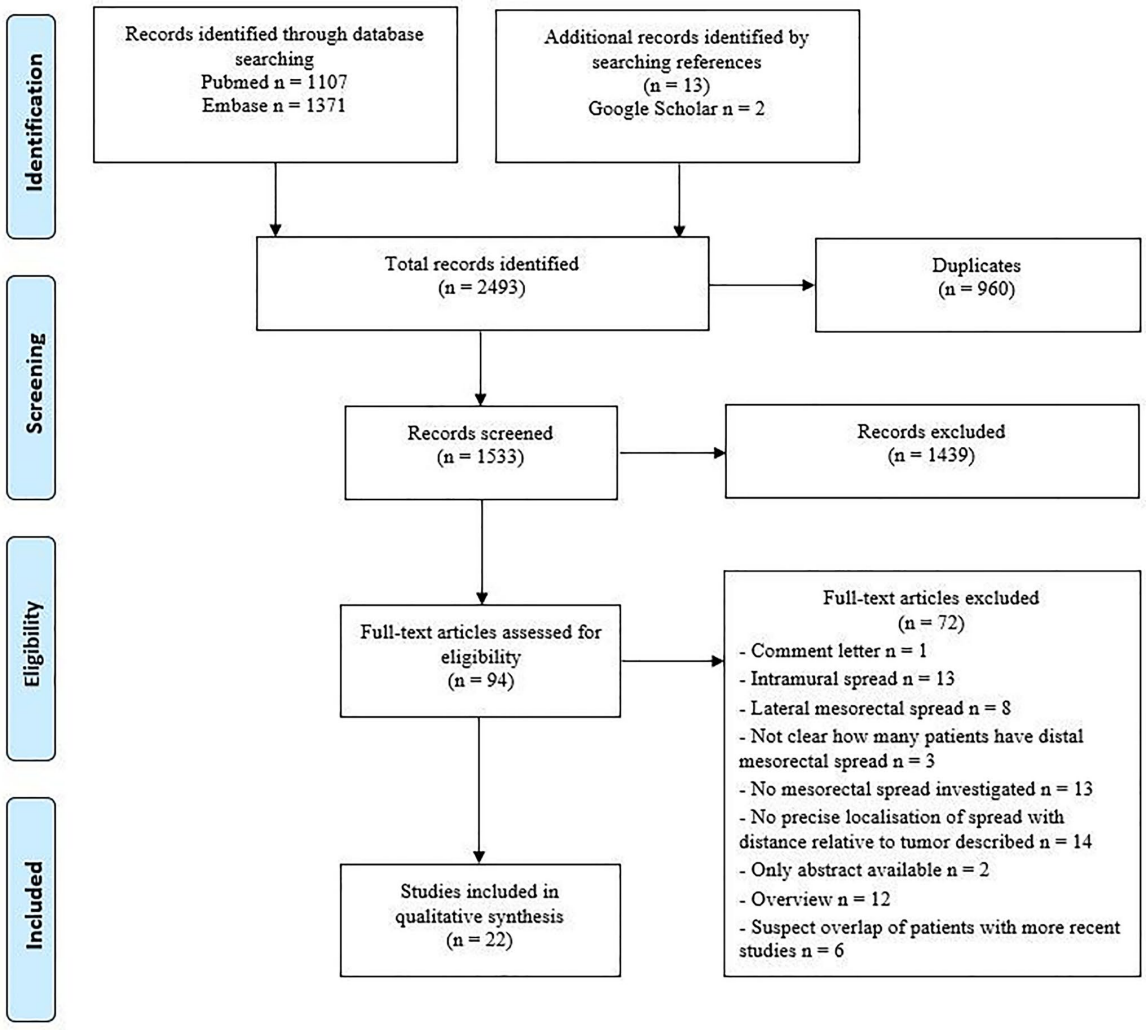
Flowchart of the literature search

The literature search yielded 2493 records that resulted in 1533 unique articles after removal of duplicates. Thirteen studies were identified by crosschecking references of the included articles. After screening on title and abstract, 94 articles were assessed by full text. A total of 72 articles were excluded by reasons outlined in Fig. 1 [1, 18–88]. The 22 included studies reported a total number of 1921 patients [89–110]. Study design and characteristics are described in Table 3. Three studies were of retrospective design [94, 95, 103], twelve of prospective design [89, 90, 92, 96, 98–101, 104, 105, 109, 110], and of the remaining seven studies, the design was unclear [91, 93, 97, 102, 106–108].

**Table 3:** Characteristics of the included studies

Study	Design	Total number of patients	Patients received long-course CRT (%)	Patients without long-course CRT with DMS of any extent (%)	Patients with CRT with DMS of any extent (%)	Mean DMS (mm)	Maximum DMS (mm)	Mode of spread (LN/TD/EMVI/DI/LP)
Choi [89]	Prospective	53	NM	11 (20.8%)	NA	NA	NM	LN and TD
Girona [90]	Prospective	47	NM	5 (10.6%)	NA	NA	NM	LN
Grinnell [91]	NM	118	NM	5 (4.2%)	NA	NA	20	LN
Guedj [92]	Prospective	124	124 (100%)	NA	1 (0.8%)	NA	20	TD
Guo [93]	NM	23	0 (0%)	3 (13%)	NA	NA	NM	LN
Heijnen [94]	Retrospective	61	61 (100%)	NA	0 (0%)	NA	0	NM
Hida [95]	Retrospective	198	NM	40 (20%)	NA	20.8	40	LN
Joh [96]	Prospective	72	NM	11 (15.3%)	NA	NA	NM	LN and TD
Kiss [97]	NM	50	NM	12 (24%)	NA	NA	50	LN and TD
Koh [98]	Prospective	16	0 (0%)	0 (0%)	NA	NA	0	NM
Langman [99]	Prospective	244	74 (30.3%)	5 (2.9%)	3 (4.1%)	NA	35	LN
Ono [100]	Prospective	40	NM	3 (7.5%)	NA	14	20	LN
Scott [101]	Prospective	20	NM	4 (20%)	NA	20	30	LN
Shan [102]	NM	62	0 (0%)	15 (24%)	NA	NA	40	LN and TD and EMVI
Shimada [103]	Retrospective	381	0 (0%)	31 (8.1%)	NA	NA	38	LN and TD
Sprenger [104]	Prospective	81	81 (100%)	NA	0 (0%)	NA	0	NM
Tocchi [105]	Prospective	53	NM	15 (28.3%)	NA	NA	NM	LN and TD
Wang [106]	NM	31	0 (0%)	4 (12.9%)	NA	NA	35	NM
Wang [107]	NM	60	NM	15 (25%)	NA	NA	40	NM
Yu [108]	NM	96	0 (0%)	6 (6.3%)	NA	NA	35	LN
Zhang [109]	Prospective	46	0 (0%)	10 (21.7)	NA	15.5	40	LN and TD and EMVI
Zhao [110]	Prospective	45	0 (0%)	8 (17.8%)	NA	12.2	36	LN and DI and LP

*NM* = not mentioned, *NA*=  not applicable, *CRT*=  chemoradiotherapy, *DMS*=  distal mesorectal spread, *LN*=  lymph node, *TD*=  tumor deposit, *EMVI*=  extramural vascular invasion, *DI*=  direct invasion, *LP*=  lymphatic permeation

### Quality assessment: agency for healthcare research and quality (AHRQ) methodological checklist

The quality assessment is shown in Supplementary Table 1. The AHRQ scores ranged from 0 to 7. Thirteen of the included studies were considered to be of low quality (0–4) [90, 91, 93, 95–101, 107–109], and the remaining nine studies to be of moderate quality (5–8) [89, 92, 94, 102–106, 110]. Items 5, 7 and 9 were not applicable for any of the included studies, because in all patients, an intended radical resection was performed. Therefore, none of the patients were excluded from analysis and missing data were not handled in the analysis. In addition, item 11 was not applicable in most of the studies. Only 4 studies included patient follow-up [102, 103, 105, 110].

### Outcomes

#### Overall DMS

DMS was found in 207 of the total of 1921 examined specimens (10.8%). In 84 of 207 cases, the distance of the DMS relative to the tumor was reported. The maximum reported DMS was 50 mm, which was found in one of the evaluable 84 specimens [97].

The overall median DMS was 20.0 mm and the overall mean DMS was 20.2 mm. DMS less than 10 mm was reported in 8.3% of the examined specimens, DMS from 10 till 20 mm was reported in 38.1% of the cases, DMS from 20 till 30 mm was reported in 21.4%, DMS from 30 till 40 was reported in 22.6%, and more than 40 mm DMS was reported in 9.5% of the 84 cases (see Table 4).

**Table 4:** The amount of specimens per distance of distal mesorectal spread (DMS)

Distance of DMS	Amount of specimens
> 0 mm and < 10 mm	7
≥ 10 and < 20 mm	32
≥ 20 and < 30 mm	18
≥ 30 and < 40 mm	19
≥ 40 mm	8

#### DMS after long-course neoadjuvant chemoradiotherapy

Four of the included articles [92, 94, 99, 104] performed pathological assessment of the specimens after long-course neoadjuvant CRT and radical resection (see Table 3). In total, 340 of the 1921 included patients (17.7%) received long-course neoadjuvant CRT and DMS was found in 4 of those 340 examined specimens (1.2%). The distance was noted in only 1 of those 4 patients with DMS and it was 20 mm. Two hundred and three of the remaining 1581 patients, who did not receive long-course neoadjuvant CRT, had DMS (12.8%).

#### DMS and T-stage

**Fig. 2:**
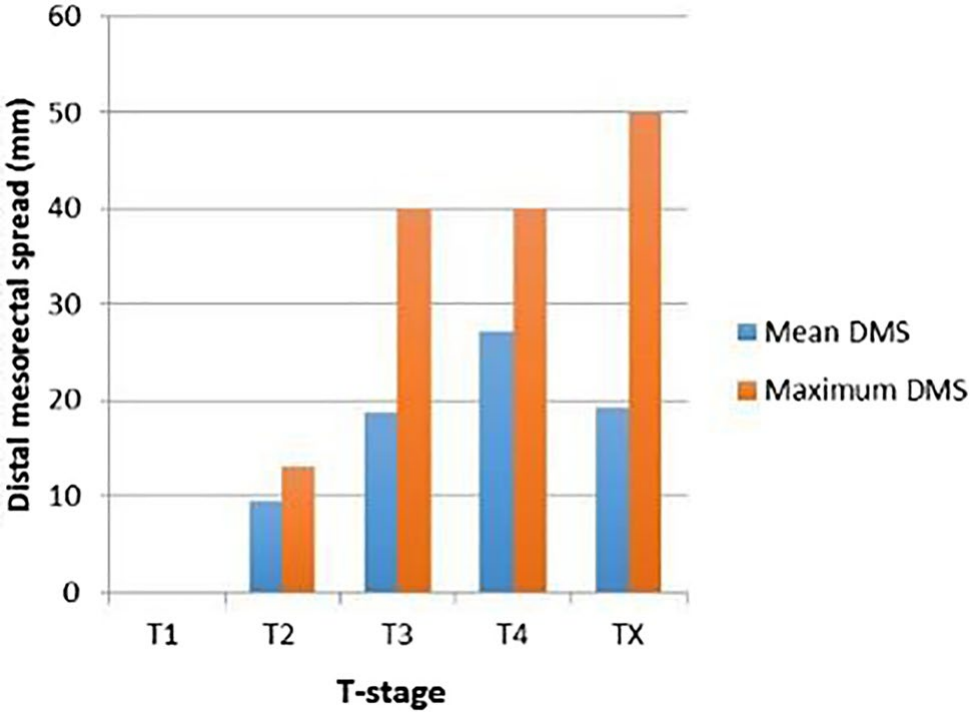
The mean and maximum distal mesorectal spread (DMS) per T-stage

Subgroup analysis for the different T categories are provided in Fig. 2 and Supplementary Fig. 1. The mean DMS for T3 rectal tumors was 18.8 mm (range: 8–40 mm), based on 36 patients. Corresponding outcome for 17 patients with T4 tumors was 27.2 mm with a range of 10–40 mm. The T-stage was not reported in 27 patients.

**Fig. 3:**
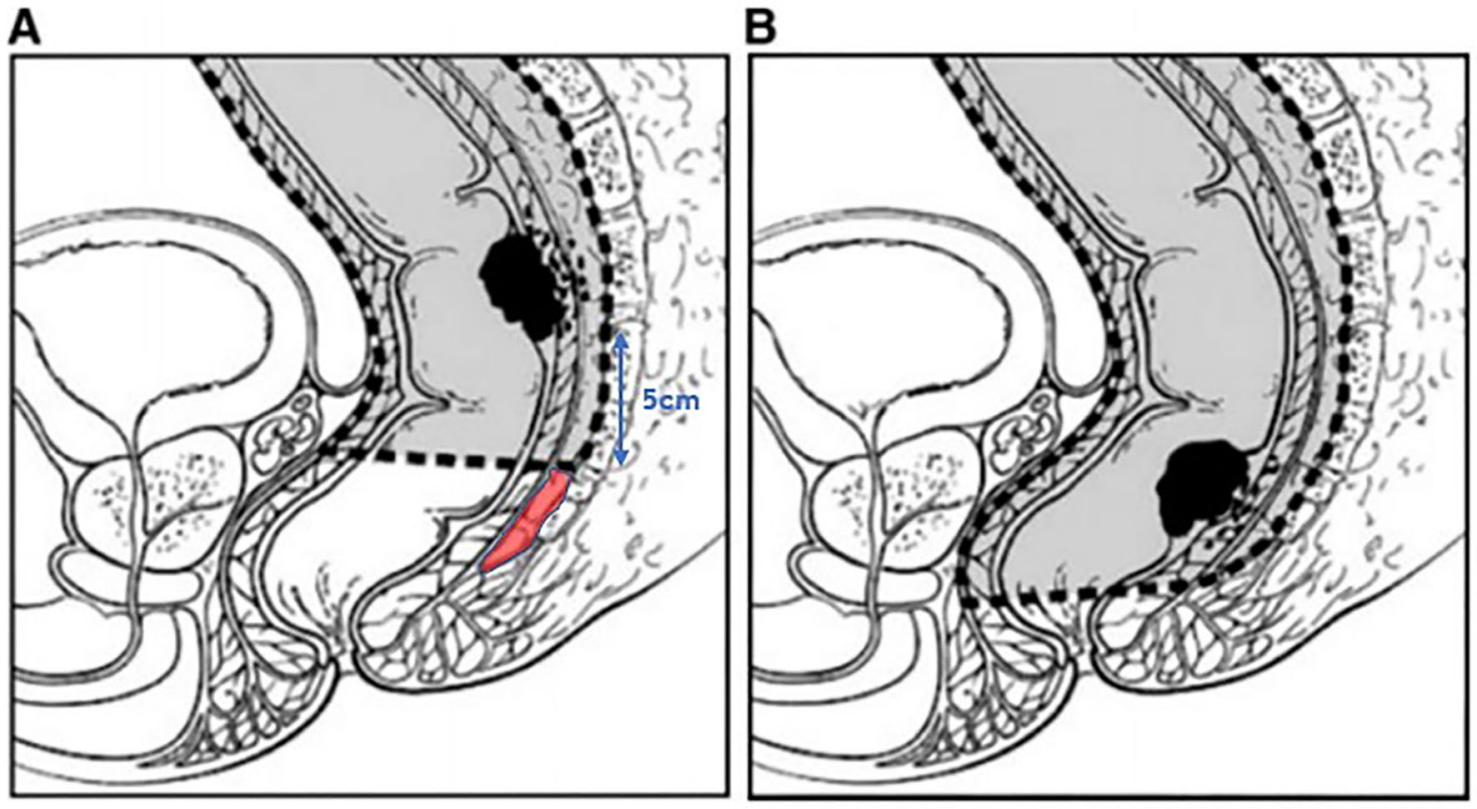
On the left side (**A**), a partial mesorectal excision is illustrated with a distal mesorectal resection margin of 5 cm. The red shaded area is residual mesorectum. On the right side (**B**), a total mesorectal excision is illustrated

#### DMS and tumor height

Results regarding high and low tumors are presented in Supplementary Figs. 2 and 3. The mean DMS in the high rectal cancer group was 22.8 mm (range: 10–40 mm). The mean DMS in the low rectal cancer group was 18.5 mm with a range of 8–30 mm.

#### DMS per T-stage and tumor height together

The mean DMS of the high located T3 and T4 rectal tumors was 23.4 mm (range: 10–40 mm). The mean DMS for the T3 and T4 tumors localized low was 18.4 mm with a range of 8–30 mm.

#### Type of DMS

Type of DMS was reported in 77 cases. In the vast majority of the cases, DMS comprised a lymph-node metastasis, namely in 65 of the 77 cases (84.4%). The mean DMS in this subgroup was 20.9 mm, with a range of 8–40 mm. In 8 cases, the spread was a tumor deposit (10.3%) with a DMS between 20 and 38 mm, in two cases, there was direct invasion distal to the mesorectum (2.6%) with a DMS of 6 and 10 mm, respectively, in one case, there was lymphatic invasion (1.3%) with a DMS of 8 mm, and in the remaining case, there was both direct invasion and a lymph-node distal in the mesorectum (1.3%) with a DMS of 10 mm.

## Discussion

To the best of our knowledge, this is the first report that has systematically reviewed the published data about DMS in rectal cancer. The incidence was 10.8%, whereas the mean reported DMS was 20.2 mm. Using a distal margin of 30 mm would have resulted in residual tumor in 32% of cases with DMS and 10% of the patients with a distal margin of 40 mm would have had residual tumor. The maximum DMS was 50 mm. However, this was only recorded in less than 1% of the specimens with DMS (1 of 84 evaluable cases). The available data suggested that DMS increased with higher T-stage. These data imply that a PME can be safely executed if a distal margin of at least 50 mm can be obtained for T3 and T4 tumors, indicating that TME should be performed for tumors located up to 5 cm proximal from the most distal part of the mesorectum as measured on preoperative MRI, see Fig. 3. This also implies that not all patients with rectal cancer based on the new consensus definition (sigmoid take-off) [111] should be treated with a formal TME. This would result in a better quality of life, since PME is associated with less morbidity and better functional outcomes than TME.

Tailoring the distal margin based on stage of disease and neoadjuvant treatment has the potential to increase the chance of tumor free distal mesorectal margins, potentially improving functional outcomes. Reducing the distal margin to 1 cm has a risk of residual DMS of 11%, which translates to an absolute risk of 1% given the fact that DMS is only found in 11% of the patients overall in this systematic review. If a distal margin of 2 cm is aimed for, a 32% risk of residual DMS results in an absolute increase of 4% for the whole group. For patients with non-locally advanced disease (T1-3a), these risks may be acceptable, given the balance between oncological safety and functional outcome. It is important to highlight that the data are not robust enough to allow firm conclusions regarding tailoring to specific patients groups, since the DMS is poorly reported.

Neoadjuvant CRT has been shown to decrease the number of (positive) lymph nodes available for pathologic assessment [112, 113]. Only 1.2% of the present examined specimens, from patients who had long-course neoadjuvant CRT and radical resection, had DMS (versus 12.8% after radical resection only). However, whether a PME with a distal margin of less than 5 cm in radiated patients is safe based on the current available data is not clear. This would require properly designed studies with a high sample size and with both DMS and local recurrence (LR) rate as endpoints.

Several studies have reported distal intramural spread in 5% of TME specimens, and this rarely exceeds 1 cm [79, 84, 110]. One cohort study from 2011 showed no significant difference in LR rates after 5 years between patients who had a distal resection margin of ≤ 1 cm and patients who had a distal resection margin of > 1 cm following TME [114]. Data from the Dutch TME trial showed that in patients with nodal disease and a distal margin of 2 cm or less, TME with radiotherapy was associated with lower recurrence rates compared to TME without radiotherapy. It was suggested that for node negative patients, a distal margin of 1 cm is sufficient, and for node positive patients, a margin of more than 2 cm is required [115].

The current review shows that DMS beyond 2 cm can occur in a proportion of patients. Individual studies showed DMS in 0–30% of patients with rectal cancer [95, 100, 105], with a pooled proportion of DMS in the present review of 10.8%. Of course, underreporting might be present, since the distal spread is not always mentioned in standard pathology reports. Furthermore, an inadequate distal mesorectal margin might result in false negative findings. Finally, there might be only 1 or 2 cm mesorectum distal to the tumor in patients with low rectal cancer, and therefore, DMS cannot occur beyond 2 cm by definition. Applying the 5 cm rule in clinical practice can be challenging. It is important to stress that the distal border of the mesorectum is often located above the level of the anorectal junction due to tapering of the mesorectum toward the pelvic floor. Surgical decision-making should be based on detailed assessment of the preoperative MRI, with extent of the resection tailored to individual anatomy. In practice, the 5 cm distal margin can be obtained in tumors located at a distance of more than 5 cm from the distal edge of the mesorectum, approximately 7 cm from the anorectal junction. Another 3 to 5 cm have to be added if the anal verge is used as a reference for tumor height, but this is less accurate and not recommended for clinical decision-making in the era of detailed preoperative staging using MRI.

Oncological safety and morbidity may be inversely related when it comes to the treatment of rectal cancer. On the one hand, morbidity should be kept as low as possible. On the other hand, treatment should be oncologically safe with the lowest chance of LR. Several centers have reported similar LR rates when TME is compared to PME with a 5 cm distal mesorectal resection margin (excluding patients receiving neoadjuvant therapy) [9, 116–118]. However, studies published from 2010 to 2015 demonstrate a concerning high rate of 10–16% LR in patients with proximal rectal cancer after PME [119–124]. Bondeven et al. found that inadvertent residual mesorectal tissue was often visualized on postoperative MRI, especially after PME (63%) [119]. Moreover, they showed that the distal mesorectal resection margin after PME, measured by postoperative MRI, was less than 5 cm in 80% and less than 3 cm in 52%. These findings could be a possible explanation for the relatively high rate of local recurrence after PME. Moreover, the quality of surgery was questionable and probably the major reason for local recurrence. Subsequently, Bondeven and co-workers investigated the impact of a multidisciplinary training program on outcomes of high rectal cancer by critical appraisal of the extent of mesorectal excision on postoperative MRI in another study [125]. The 3-year LR rate fell from 12.9% to 5.0%, and none of the patients treated with PME developed local recurrence when a distal resection margin of at least 3.5 cm was achieved. This illustrates the importance of a good quality of distal mesorectal excision and demonstrates that local recurrence is comparable to TME when PME is performed with adequate margins.

A good quality of mesorectal excision is not only about the distal mesorectal resection margin. Some studies suggest that the integrity of the surgical plane is more important. Jiménez-Toscano et al. recently found no significant difference in local-recurrence-free survival, DFS and OS between patients with ≤ 10 mm, 11–20 mm, 21–30 mm or ≥ 31 mm distal mesorectal resection margin [126]. In agreement with Quirke et al., classification of the integrity of the planes together with stage of disease were the most important factors for LR rates [127].

This systematic review has several limitations. First, there are paucity of data in the literature and this combined with the heterogeneity and lack of detail in the existing data in terms of T-stage and neoadjuvant therapy use limits the strength of the conclusions that can be drawn. In particular, DMS is not always included in datasets as an outcome, since routine pathologic examination of the entire mesorectal specimen for individual tumor cells is not standard practice. This could explain why DMS was found in only approximately 11% of the specimens. Second, postoperative MRI data from Bondeven et al. found residual mesorectum in many cases where a formal TME was intended, potentially resulting in underreporting of DMS [119, 128]. A further limitation relates to quality. More than half of the included studies were considered to be of low quality. One of the included studies is old (1950) and the surgical and histological reporting standards in that time were less accurate. Furthermore, similarly to refinements of surgical technique, neoadjuvant CRT regimens have likely been adjusted over time. Another limitation is that most of the included studies did not take into account the fact that shrinkage occurs in fixed specimens. The length of the distal resection margin may reduce by up to 30% after fixation [23, 129]. Bearing in mind that most of the included studies investigated fixed specimens, the DMS might potentially be higher than reported. In addition, inter-observer variability between pathologists can occur, as is shown in the study of Mekenkamp et al. [130]. Finally, the included studies did not report long-term oncological outcomes such as local recurrence..

These limitations mean that definite indications about the distal mesorectal resection margin should be in PME, and when a PME is safe or when a TME should be performed, it is not possible. Clearly, there is a need for a properly designed and quality controlled (international) study to prospectively evaluate the incidence of distal mesorectal tumor spread by precise pathologic assessment, with and without neoadjuvant treatment. This could be combined with a modified Delphi study to determine a core outcome set for pathology and MRI. In the meantime, we recommend a detailed assessment of the preoperative MRI taking into account individual variability in anatomy of the distal mesorectum, combined with a discussion of the balance between oncological safety and functional outcome with the patient, to guide the decision on the level of mesorectal transection in rectal cancer.

## Conclusions

This systematic review shows that PME is a safe procedure in those patients where a margin of 5 cm can be obtained. The data revealed an incidence of DMS in rectal cancer of 11% overall, which was 1% and 13% with and without long-course neoadjuvant CRT. The maximum reported DMS was 50 mm based on a single case, with a risk of residual DMS of 1% using a 40 mm distal margin and this risk increases to 4% with a 30 mm distal margin. Prospective studies evaluating margins based on high-quality preoperative MRI and pathological assessment are required.

## Supplementary Information

Below is the link to the electronic supplementary material.

Supplementary Figure 1 Scatter plot with the individual patients with distal mesorectal spread (DMS) per T-stage. Supplementary file1 Scatter plot with the individual patients with distal mesorectal spread (DMS) per T-stage (JPG 40 KB)

Supplementary Figure 2 The mean and maximum distal mesorectal spread (DMS) per level of rectal tumor Supplementary file2 The mean and maximum distal mesorectal spread (DMS) per level of rectal tumor (JPG 61 KB)

Supplementary Figure 3 Scatter plot with the individual patients with distal mesorectal spread (DMS) per level of rectal tumor. Supplementary file3 Scatter plot with the individual patients with distal mesorectal spread (DMS) per level of rectal tumor (JPG 54 KB)

Supplementary file4 (DOCX 22 KB)

## Data Availability

The data that support the findings of this study are available from the corresponding author upon reasonable request.
