# Supplementary figures and images for: Required distal mesorectal resection margin in partial mesorectal excision: a systematic review on distal mesorectal spread
Source: Tech Coloproctol. 2022 Aug 29;27(1):11–21. doi: 10.1007/s10151-022-02690-1 (PMC9807492; doi:10.1007/s10151-022-02690-1)

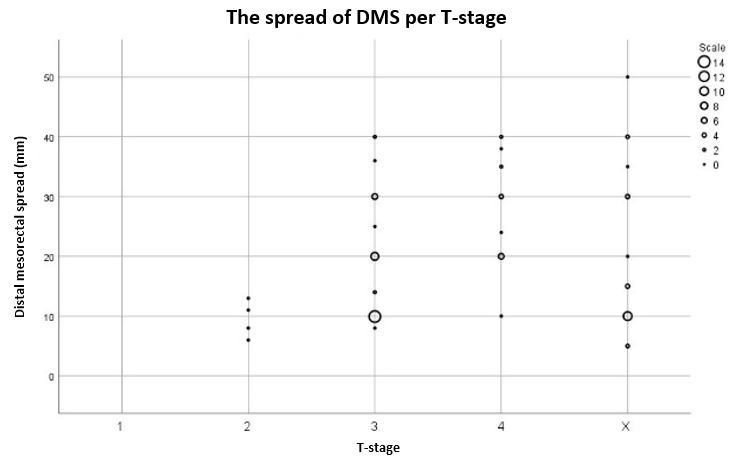

Supplement: Supplementary Figure 1 — Scatter plot with the individual patients with distal mesorectal spread (DMS) per T-stage. [file 10151_2022_2690_MOESM1_ESM.jpg]

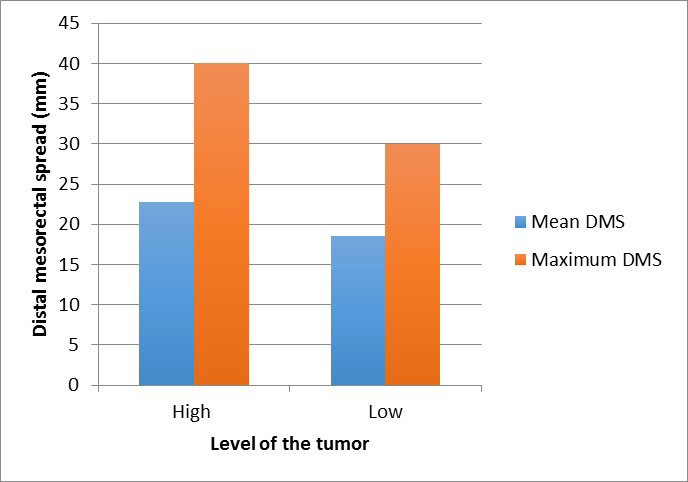

Supplement: Supplementary Figure 2 — The mean and maximum distal mesorectal spread (DMS) per level of rectal tumor [file 10151_2022_2690_MOESM2_ESM.jpg]

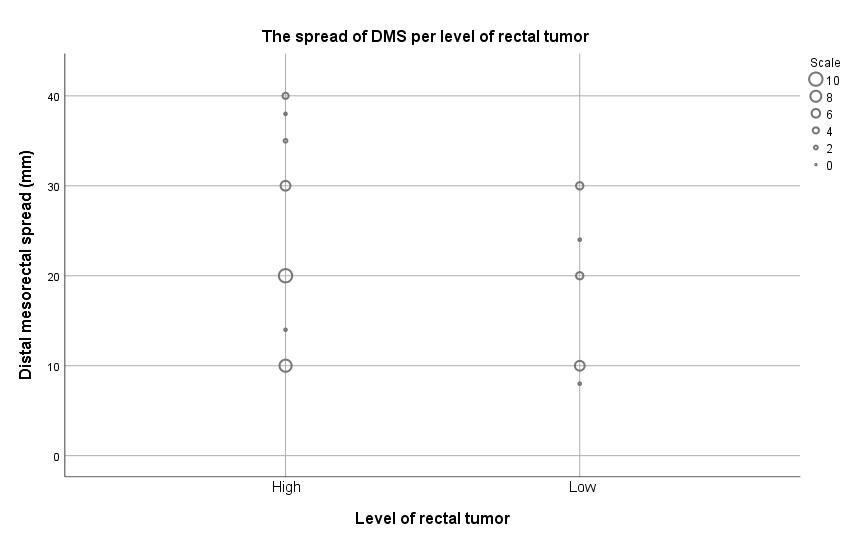

Supplement: Supplementary Figure 3 — Scatter plot with the individual patients with distal mesorectal spread (DMS) per level of rectal tumor. [file 10151_2022_2690_MOESM3_ESM.jpg]
