# Supplementary material for: Required distal mesorectal resection margin in partial mesorectal excision: a systematic review on distal mesorectal spread
Source: Tech Coloproctol. 2022 Aug 29;27(1):11–21. doi: 10.1007/s10151-022-02690-1 (PMC9807492; doi:10.1007/s10151-022-02690-1)
Supplement: Supplementary file 4 — Supplementary file4 (DOCX 22 KB) [file 10151_2022_2690_MOESM4_ESM.docx]

**Supplementary Table 1** Agency for Healthcare Research and Quality (AHRQ) checklist to asses quality of the included studies.

| ***AHRQ checklist items*** | **Choi (1996)** | **Girona (1993)** | **Grinnell (1949)** | **Guedj (2016)** | **Guo (2010)** | **Heijnen (2016)** | **Hida (1997)** | **Joh (1999)** | **Kiss (2011)** | **Koh (2005)** | **Langman (2015)** | **Ono (2002)** | **Scott (1995)** | **Shan (2019)** | **Shimada (2011)** | **Sprenger (2013)** | **Tocchi (2001)** | **Wang (2005)** | **Wang (2008)** | **Yu (2011)** | **Zhang (2008)** | **Zhao (2005)** |
| --- | --- | --- | --- | --- | --- | --- | --- | --- | --- | --- | --- | --- | --- | --- | --- | --- | --- | --- | --- | --- | --- | --- |
| 1. Define source of information (survey, record review) | + | + | + | + | + | + | + | + | + | + | + | + | + | + | + | + | + | + | + | + | + | + |
| 2. List inclusion and exclusion criteria for exposed and unexposed subjects (cases and controls) or refer to previous publications | + | + | - | + | - | + | - | + | - | - | - | - | - | + | + | + | - | - | + | - | - | - |
| 3. Indicate time period used for identifying patients | + | + | - | + | + | + | + | + | - | - | + | + | - | + | + | + | + | + | + | + | + | + |
| 4. Indicate whether or not subjects were consecutive if not population-based | + | U | U | + | U | + | + | U | U | U | U | + | U | U | + | U | + | + | - | U | U | + |
| 5. Indicate if evaluators of subjective components of study were masked to other aspects of the status of the participants | NA | NA | NA | NA | NA | NA | NA | NA | NA | NA | NA | NA | NA | NA | NA | NA | NA | NA | NA | NA | NA | NA |
| 6. Describe any assessments undertaken for quality assurance purposes (e.g., test/retest of primary outcome measurements) | + | - | - | - | + | U | - | U | + | + | + | - | - | + | + | + | + | + | - | + | + | + |
| 7. Explain any patient exclusions from analysis | NA | NA | NA | NA | NA | NA | NA | NA | NA | NA | NA | NA | NA | NA | NA | NA | NA | NA | NA | NA | NA | NA |
| 8. Describe how confounding was assessed and/or controlled | - | - | - | - | - | - | - | - | - | - | - | - | - | - | - | - | - | - | - | - | - | - |
| 9. If applicable, explain how missing data were handled in the analysis | NA | NA | NA | NA | NA | NA | NA | NA | NA | NA | NA | NA | NA | NA | NA | NA | NA | NA | NA | NA | NA | NA |
| 10. Summarize patient response rates and completeness of data collection | + | + | - | + | + | + | + | - | - | + | + | + | + | - | + | + | - | + | + | - | - | + |
| 11. Clarify what follow-up, if any, was expected and the percentage of patients for which incomplete data or follow-up was obtained | NA | NA | NA | NA | NA | NA | NA | NA | NA | NA | NA | NA | NA | + | + | NA | + | NA | NA | NA | NA | + |
| **Total points** | 6 | 4 | 1 | 5 | 4 | 5 | 4 | 3 | 2 | 3 | 4 | 4 | 2 | 5 | 7 | 5 | 5 | 5 | 4 | 3 | 3 | 6 |

*+ = Yes; - = No; U = Unclear; NA = not applicable.*
